# A hybrid combination of *in vitro* cultured buccal mucosal cells using two different methodologies, complementing each other in successfully repairing a stricture-inflicted human male urethral epithelium

**DOI:** 10.3389/fruro.2025.1720445

**Published:** 2026-01-14

**Authors:** Akio Horiguchi, Toshihiro Kushibiki, Yoshine Mayumi, Masayuki Shinchi, Kenichiro Ojima, Yusuke Hirano, Shojiro Katoh, Masaru Iwasaki, Vaddi Surya Prakash, Koji Ichiyama, Rajappa Senthilkumar, Senthilkumar Preethy, Samuel J. K. Abraham

**Affiliations:** 1 Division of Reconstruction, Center for Trauma, Burn and Tactical Medicine, National Defence Medical College, Tokorozawa, Saitama, Japan; 2 Department of Medical Engineering, National Defence Medical College, Tokorozawa, Saitama, Japan; 3 Department of Orthopedics, Edogawa Hospital, Edogawa, Japan; 4 Center for Advancing Clinical Research (CACR), Faculty of Medicine, University of Yamanashi, Chuo, Japan; 5 Department of Urology, Surya Kidney Centre, Hyderabad, India; 6 Department of Urology, Kamineni Academy of Medical Sciences and Research Centre, Hyderabad, India; 7 Antony- Xavier Interdisciplinary Scholastics (AXIS), GN Corporation Co. Ltd., Kofu, Japan; 8 R&D Division, JBM Inc., Edogawa, Japan; 9 The Fujio-Eiji Academic Terrain (FEAT), Nichi-In Centre for Regenerative Medicine (NCRM), Chennai, India; 10 The Mary-Yoshio Translational Hexagon (MYTH)-NCRM, MediNippon Healthcare Pvt. Ltd., Chennai, India; 11 II Department of Surgery, University of Yamanashi, Chuo, Japan; 12 Levy-Jurgen Transdisciplinary Exploratory (LJTE), Global Niche Corp., Wilmington, DE, United States; 13 Surya Akio Horiguchi Lab for Tissue Engineering (SALT), SoulSynergy Ltd., Phoenix, Mauritius

**Keywords:** BEES-HAUS, buccal mucosa, epithelium, hybrid culture, IGF-1, regenerative medicine, cell therapy, urethral stricture

## Abstract

**Background:**

Autologous buccal mucosa cell transplantation has emerged as a promising treatment strategy for urethral stricture disease. However, ambiguity has persisted regarding the optimal cell type and culture conditions that aid successful urethral repair. Clinical study of our previously reported cell-based endoscopic approach, the buccal epithelium expanded and encapsulated in scaffold-hybrid approach to urethral stricture (BEES-HAUS), demonstrated durable epithelial regeneration and long-term urethral patency. The present work provides mechanistic insights supporting the BEES-HAUS approach of combining two-dimensional (2D) monolayer-cultured fibroblast-like cells and three-dimensional (3D) thermo-responsive gelation polymer (Festigel)-cultured cells.

**Methods:**

Human buccal tissues (n=22) were cultured in two methods; one portion using the monolayer method (2D), and the other in 3D using Festigel. Flow cytometry for phenotype markers and ELISA for IGF-1 were carried out.

**Results:**

3D Festigel-cultured cells acquired an epithelial phenotype, with AE1/AE3 expression up to day 21, while 2D cultures yielded fibroblast-like CD140b-positive/AE1-AE3–negative cells. IGF-1 secretion was significantly higher in 2D cultures than 3D Festigel (p < 0.05), indicating a supportive paracrine role. These findings explain the complementary contribution of epithelial integration and IGF-1–mediated support observed as successful clinical outcome of the BEES-HAUS procedure.

**Conclusion:**

This study, a first of its kind, clarifies the rationale and advantages of combining 3D Festigel-expanded epithelial cells with the paracrine effect of IGF-1-secreting 2D fibroblast-like cells in a single transplantation strategy, thereby explaining the successful clinical outcomes reported in BEES-HAUS. Further research on this hybrid cell combination is recommended to expand this approach for regenerating and repairing other tissues and organs.

## Graphical Abstract

**Figure:**
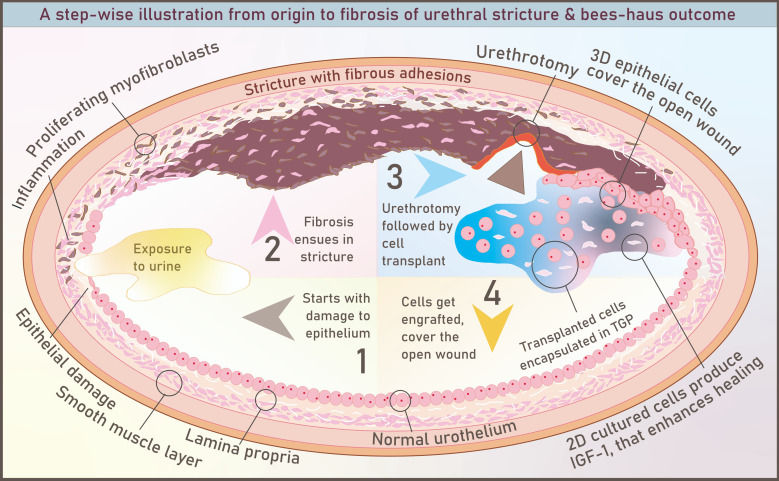
Illustration of the pathogenesis of urethral stricture and the contribution of the hybrid combination of two-dimensional (2D) and three-dimensional (3D-Festigel) cultured cells to the successful repair of the stricture-inflicted male urethral epithelium in the buccal epithelium expanded and encapsulated in scaffold-hybrid approach to urethral stricture (BEES-HAUS) technique.

## Introduction

Urethral stricture disease (USD) is characterized by a narrowing of the urethral lumen resulting from ischemic spongiofibrosis. Commonly postulated etiological factors include trauma from urethral instrumentation, infections, and inflammatory conditions (1). These factors lead to epithelial injury followed by healing through fibrosis, ultimately reducing the size of the urethral lumen, impairing urinary flow and potentially affecting reproductive function (2). Conventional management strategies for USD such as urethral dilatation, internal urethrotomy, and urethroplasty along with pharmacological approaches including anti-fibrotic agents and growth hormones, have been explored. However, these treatments may introduce additional morbidity and are frequently associated with stricture recurrence, limiting their long-term therapeutic effectiveness (1).

Recent advances have expanded interest beyond surgical and pharmacological therapies toward cellular and non-cellular regenerative approaches. Submucosal injection of platelet-rich plasma (PRP) following direct visual internal urethrotomy (DVIU) has shown encouraging outcomes in reducing recurrence in patients with primary, short bulbar strictures (3). Mesenchymal stem cell (MSC)–based therapies have also been investigated for their regenerative potential in USD (1). Tissue-engineering strategies incorporating synthetic or natural biomaterial scaffolds with cells and bioactive molecules have been clinically reported, including autologous tissue-engineered buccal mucosa (TEBM) (4). Furthermore, regulatory-approved tissue-engineering products are already available in select European countries (5, 6). We developed a novel cell-based endoscopic technique, the Buccal Epithelium Expanded and Encapsulated in Scaffold-Hybrid Approach to Urethral Stricture (BEES-HAUS) which demonstrated successful outcomes in a clinical study involving six patients (7). Following the BEES–HAUS procedure, all patients achieved normal voiding with an average peak flow rate of 24 mL/s, and six-month urethroscopy confirmed healthy mucosal regeneration at the urethrotomy site. A subsequent rabbit study provided proof of engraftment of transplanted buccal mucosal cells over the urothelium at the urethrotomy site (8, 9).

In the BEES-HAUS procedure, a combination of two-dimensional (2D) monolayer-cultured cells and three-dimensional (3D) Festigel-cultured cells is utilized and delivered using Festigel as the carrier scaffold (7–9). The present study aims to evaluate the advantages of this 2D and 3D cell combination used for transplantation in the BEES-HAUS technique (7–9). Specifically, we assess IGF-1 secretion and AE1/AE3 pancytokeratin expression in cultured cells prior to transplantation, thereby providing technical clarification of their complementary contributions to the observed clinical success.

## Methods

The study was conducted in accordance with the Declaration of Helsinki and relevant institutional and national guidelines. Ethical approval was obtained from the Ethics Committee of the National Defence Medical College, Japan (Approval number: 4154; dated 9 April 2020). Human buccal tissue samples (n = 22) were collected from adult patients undergoing biopsy for buccal mucosal graft urethroplasty. Tissues rendered redundant after surgery were used for experimentation with informed consent.

Samples were transported in Festigel (Free-from-Endotoxin-excess-Scaffold of Thermoresponsive Intelli-GEL), provided by M/s GN Corporation, Japan. For transport and culture preparation, Festigel was reconstituted with 10 mL of Dulbecco’s Modified Eagle Medium (DMEM)/F12 (Gibco BRL, Gaithersburg, MD, USA) and stored at 4°C until use.

Buccal tissues were subjected to enzymatic digestion using 1 mL of digestion medium containing 1,000 PU/mL Dispase I (Oenon, Japan) in DMEM (Invitrogen) and incubated overnight at 37 °C. The epithelial layer was then separated, minced, and further digested with 0.5 mL Accutase (Sigma) for 15 minutes at 37 °C. The resulting cell suspension was washed twice by centrifugation at 1500 rpm for 10 minutes. Cell viability and counts were assessed using the trypan blue exclusion method. Each sample was divided into two equal portions for culture.

In Group I (2D), cells were seeded into 12-well tissue culture plates (Greiner Bio-One, Austria) and maintained in culture medium supplemented with 10% autologous serum. In Group II (3D-Festigel), cells were mixed with reconstituted, cold-liquefied Festigel and dispensed into 12-well plates under cold conditions. The mixture was allowed to solidify for 1 minute, after which medium containing 10% autologous serum was added. Both culture groups were maintained at 37°C in a humidified atmosphere with 5% CO_2_ for up to 14–21 days.

After the stipulated days of culture, cells from both 2D and 3D-Festigel cultures were harvested following protocols established by Vaddi et al. (7), Horiguchi et al. (8, 9) and Katoh et al. (10). Harvested cells underwent histological evaluation, flow cytometric analysis (AE1/AE3 and CD140b), and the corresponding culture supernatants were collected for IGF-1 quantification by ELISA.

Hematoxylin and eosin (H&E) staining was performed using standard protocols. For flow cytometry, cells were centrifuged and stained with isotype controls and antigen-specific antibodies before acquisition on a FACSVia™ cell analyzer (BD Biosciences). Data were processed using FlowJo software (BD Biosciences).

### Antibodies used

- PE-AE1/AE3 (NSJ Bioreagents; Cat# V2330PE-100T)
- FITC-CD31 (BioLegend; Cat# 303103)
- PerCP/Cy5.5-CD326 (BioLegend; Cat# 369803)
- APC-CD140b (BioLegend; Cat# 323608)

Isotype control antibodies were obtained from BioLegend and BD.

IGF-1 levels in culture supernatants were measured using the Human IGF-I/IGF-1 Quantikine ELISA Kit (R&D Systems; Cat# DG100B), following the manufacturer’s instructions.

Statistical analyses were performed using Microsoft Excel and GraphPad Prism. Comparisons between groups were conducted using the t-test. Statistical significance was defined as *p* < 0.05. Data are presented as mean ± standard deviation (SD).

## Results

**Figure 1:**
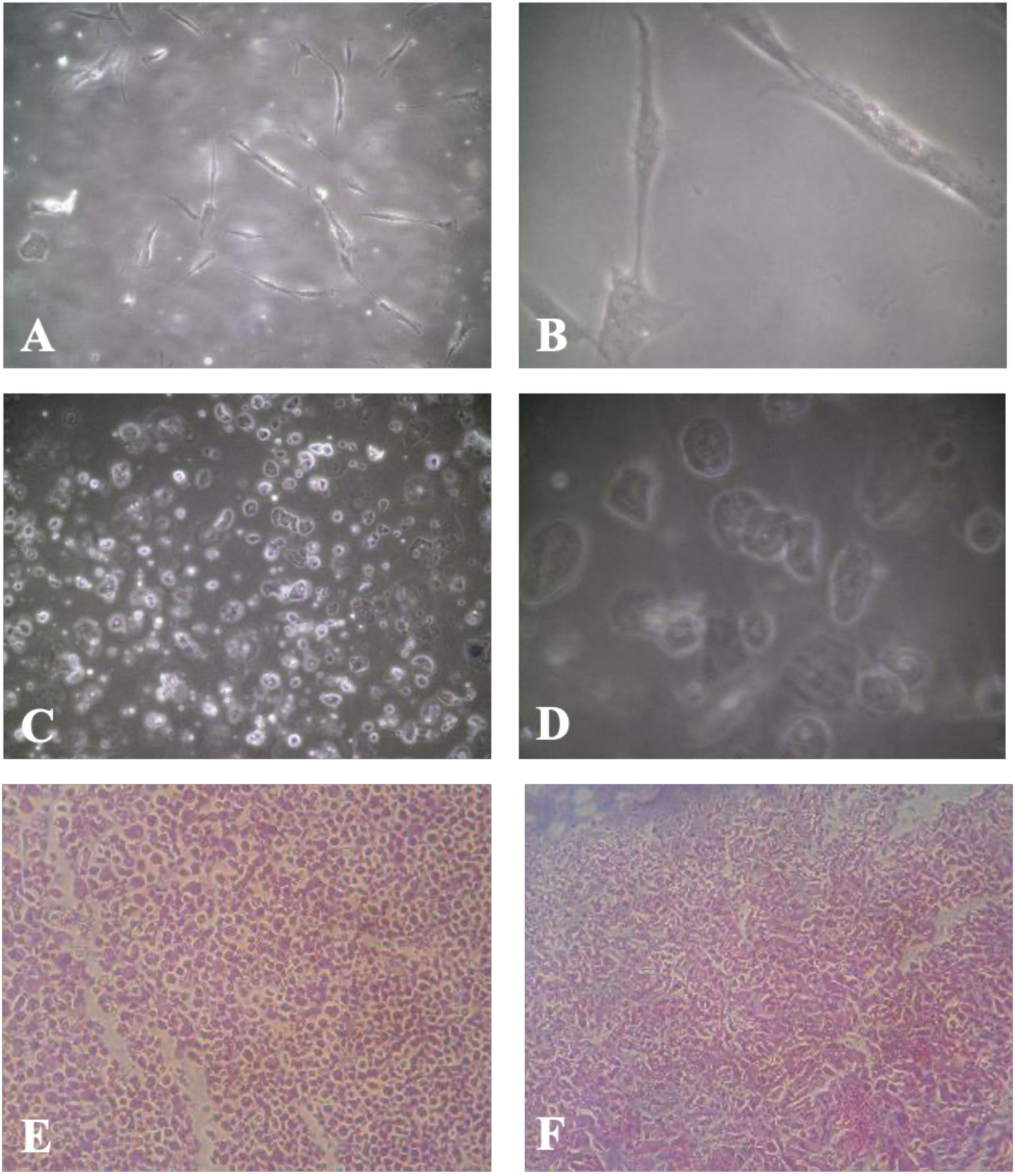
Representative culture morphology of 2D and 3D-Festigel. **(A)** 2D monolayer culture (passage 2) at ×10 magnification showing spindle-shaped fibroblast-like cells. **(B)** 2D culture (passage 2) at ×40 magnification demonstrating dispersed individual cells. **(C)** 3D-Festigel culture at ×10 magnification showing epithelial-like cell organization (no passage was performed prior to imaging). **(D)** 3D-Festigel culture at ×40 magnification displaying multilayered epithelial morphology. **(E, F)** Hematoxylin and eosin (H&E) staining performed after cell harvest: **(E)** 2D cultures showing isolated cells and **(F)** 3D-Festigel cultures demonstrating continuous tissue-like architecture.

The initial cell yield from buccal biopsies was 3.16 ± 2.51 × 10^6^ cells. After culture, 3D Festigel demonstrated significantly higher expansion (1.84 ± 1.50 × 10^6^ cells) compared with 2D monolayer (0.72 ± 0.86 × 10^6^ cells, p = 0.004). Across all 22 samples, 3D cultures showed consistent proliferation, whereas 2D cultures displayed highly variable and generally limited cell proliferation (**Supplementary Table S1**). The 2D cultures shown in **Figure 1** were imaged at passage 2, whereas 3D-Festigel cultures were examined without passaging. After 14–21 days, H&E staining of harvested cells demonstrated isolated fibroblast-like cells in 2D cultures and a continuous tissue-like epithelial structure in 3D-Festigel (**Figure 1**).

**Figure 2:**
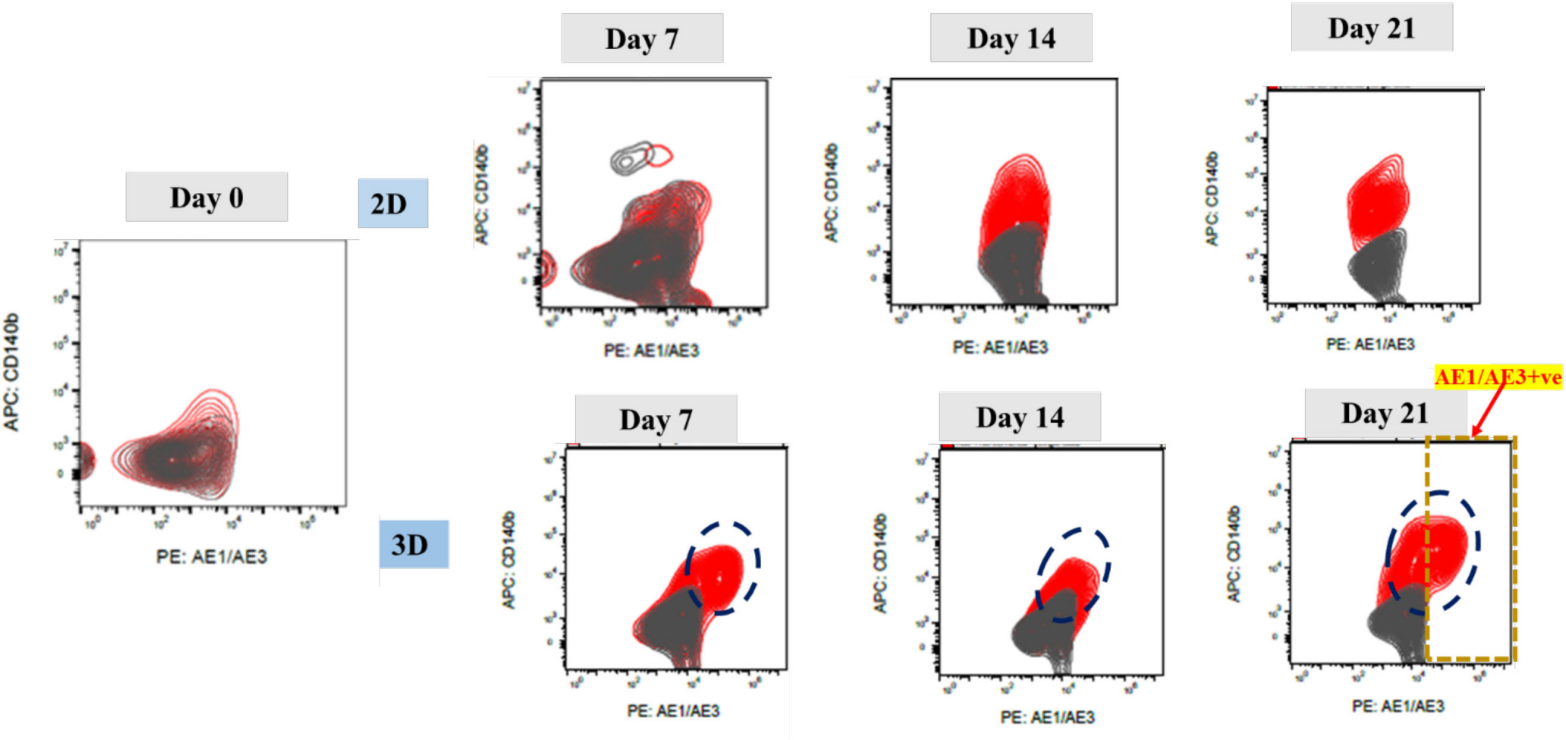
Flow cytometry analysis showing progressive increase in epithelial marker expression in 3D Festigel cultures. Cells were stained with AE1/AE3 (PE-conjugated pancytokeratin) and CD140b (APC-conjugated). AE1/AE3 positivity progressively increased from day 7 to day 21 in 3D culture, whereas 2D cultures remained AE1/AE3-negative and CD140b-positive, indicative of a fibroblast-like phenotype.

**Figure 3:**
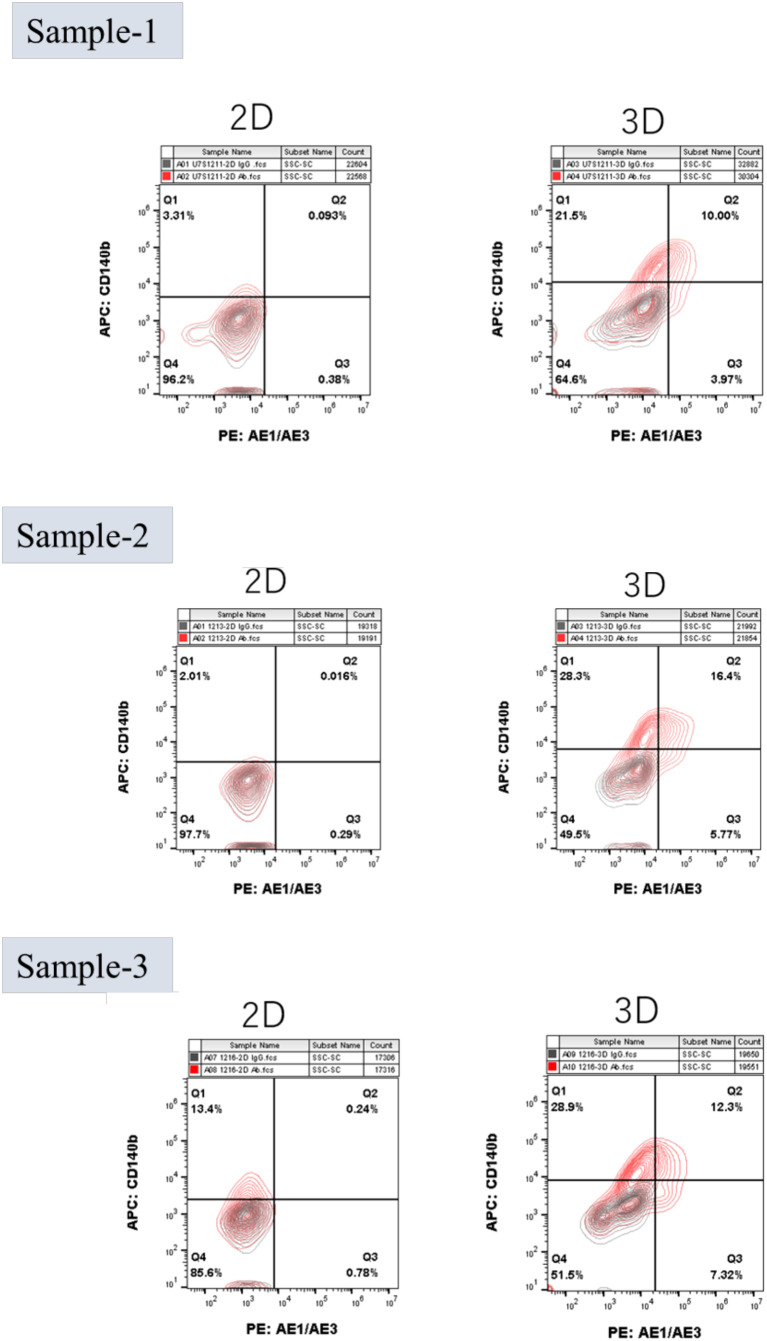
Flow cytometry plots from three representative patient samples (n = 3 of 22) comparing 2D and 3D culture conditions. 3D Festigel cultures show increased AE1/AE3 expression, while 2D cultures remain AE1/AE3-negative and CD140b-positive. Full dataset for all 22 samples is provided in **Supplementary Table S1**.

**Figure 4:**
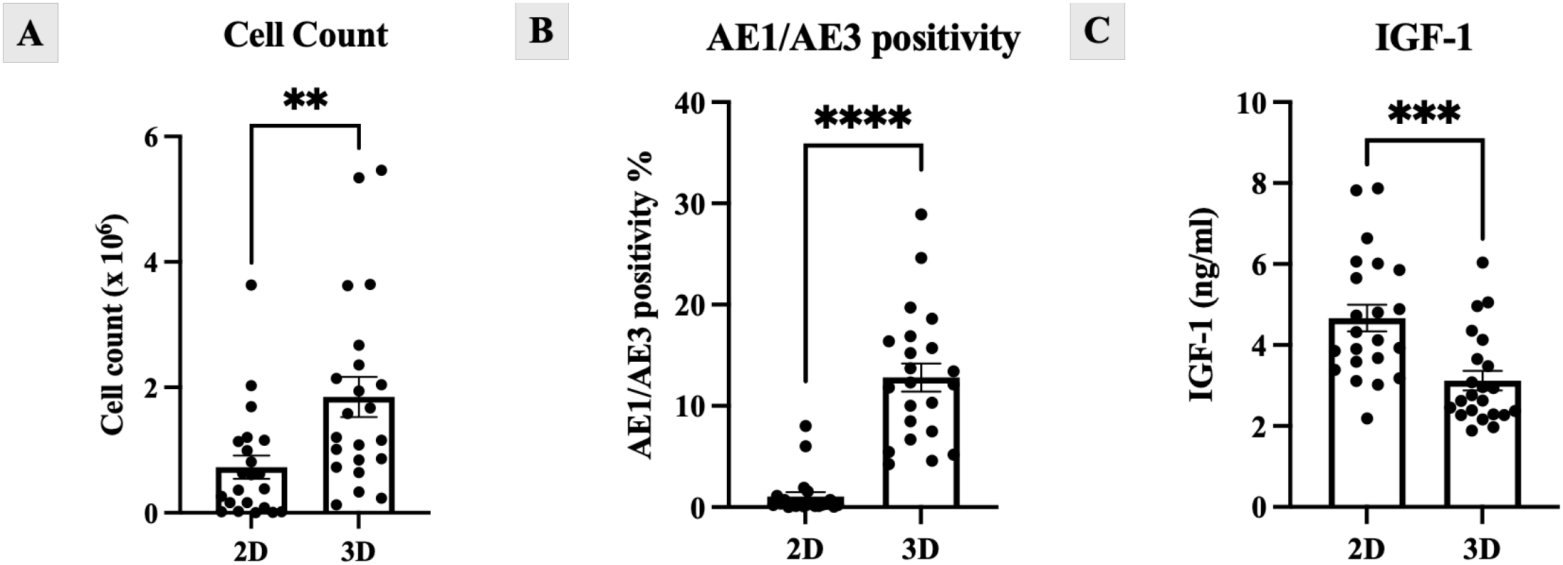
Quantitative comparison of 2D and 3D culture conditions. 3D Festigel cultures demonstrated **(A)** higher final cell expansion and **(B)** increased AE1/AE3 epithelial marker expression, while 2D monolayer cultures showed **(C)** significantly higher IGF-1 secretion into the supernatant.

Flow cytometry demonstrated progressive acquisition of an epithelial phenotype in 3D cultures with AE1/AE3 positivity increasing over time, reaching 12.81 ± 6.46% at day 21, compared with 1.04 ± 2.01% in 2D (p < 0.0001). In contrast, 2D cultures remained AE1/AE3-negative/CD140b-positive, consistent with a fibroblast-like profile (**Figures 2**, **3**, **4**). ELISA of culture supernatants showed significantly higher IGF-1 secretion in 2D (4.64 ± 1.53 ng/mL) versus 3D (3.12 ± 1.12 ng/mL, p = 0.0005), suggesting fibroblast-like cells (**Figure 4**).

## Discussion

**Figure 5:**
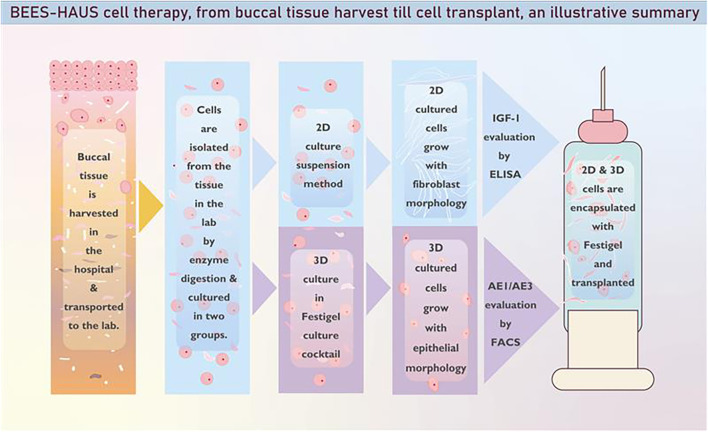
Schematic representation of the Buccal Epithelium Expanded and Encapsulated in Scaffold-Hybrid Approach to Urethral Stricture (BEES-HAUS). In this technique, 2D monolayer-cultured cells and 3D-Festigel–cultured cells are combined and transplanted using Festigel, a thermo-reversible gelation polymer (TGP), as the carrier scaffold.

The BEES-HAUS approach represents a unique, first-of-its-kind reported clinical application in which autologous buccal mucosal epithelial cells expanded using both two-dimensional (2D) monolayer and three-dimensional (3D) Festigel culture methods were transplanted together (**Figure 5**), resulting in successful epithelial restoration and long-term stricture-free patency in human patients (7–9) with engraftment of the transplanted cells holding potential for preventing recurrence (8, 11). The present findings provide technical clarity supporting this clinical observation. The higher AE1/AE3 expression in 3D-Festigel cultures confirms preservation of epithelial phenotype, while 2D-cultured cells retained a CD140b-positive fibroblast-like profile, indicating a complementary biological role during repair.

Regenerative cell therapies may facilitate healing either through direct cell integration or by modulating molecular pathways that support tissue repair (12). Increasing evidence suggests that the primary mechanism of benefit is paracrine-mediated regulation of inflammation, angiogenesis, and matrix remodeling (12). In this context, the present study is the first of its kind which demonstrates the mechanistic insights into the tissue regeneration process wherein both paracrine contribution from 2D-cultured cells and epithelial integration from 3D-Festigel–expanded cells within the host urothelium would have contributed to the successful clinical outcome observed in BEES-HAUS (7–9). IGF-1 is of particular relevance as it enhances keratinocyte migration and proliferation and has been shown to prevent urethral stricture formation in experimental models (13, 14). The significantly higher IGF-1 secretion observed in 2D cultures in the present study provides a mechanistic explanation for the stromal support achieved during BEES-HAUS, complementing the epithelial contribution from 3D Festigel-cultured cells.

The rationale for combining both culture conditions was two-fold: to maximize the number of autologous cells available for transplantation, as higher cell dose has been associated with improved engraftment (15), and to preserve epithelial characteristics in the absence of feeder layers. Festigel provides a 3D environment that supports epithelial maintenance (16), while fibroblast-like cells in 2D may contribute regenerative cytokines including interleukins, transforming growth factors, platelet-derived growth factor, and epithelial-specific mitogens such as keratinocyte and epidermal growth factors (17). Together, these findings support the concept that epithelial–mesenchymal interactions play a critical role in urethral mucosal regeneration (18, 19).

The current results also strengthen previous clinical and pre-clinical BEES-HAUS evidence (7–9) by demonstrating that the same hybrid culture system *in vitro* yields cells with distinct but complementary functions which continue the regeneration process by proliferation and migration *in vivo* as well after transplantation. Further studies are required to define the molecular signaling events involved and to evaluate applicability in other epithelial repair settings, such as esophageal or intestinal mucosal injury.

The Festigel scaffold used in BEES-HAUS has previously supported the culture or delivery of multiple cell types and tissues (20–26) and has enabled cell transportation without cold chain and scaffold-based transplantation in several pre-clinical and clinical settings (27–30), including urethral stricture repair (7–9). In our study, 3D-Festigel likely facilitated post-transplant cell migration, consistent with prior documentation in corneal epithelial models (31). Notably, Festigel does not support fibroblast proliferation *in vitro*, which, *in vivo*, may contribute to reduced fibrosis but this potential anti-fibrotic effect requires further investigation. Unlike prior tissue-engineered urethral constructs requiring pre-formed multilayered grafts, such as those reported by Atala et al. using acellular collagen matrices (32), BEES-HAUS achieves *in vivo* epithelialization and continued proliferation post-transplantation without ex vivo tissue assembly. Similarly, approaches such as in-body biotube construction (33) have demonstrated feasibility for temporary luminal replacement but show limited epithelialization and angiogenesis *in vivo*. In contrast, BEES-HAUS supports both sustained epithelial coverage and functional luminal restoration, warranting further investigation for broader clinical applications.

This study is limited by the inability to characterize the dynamic inflammatory and fibrotic environment in human strictures, as no current *in vitro* system can fully recapitulate the *in vivo* milieu. While IGF-1 was quantified, additional soluble factors may contribute to repair and require future profiling. Cell dosing also remains empirical, as the extent of pathological involvement varies across patients. Differences between chronic human disease and single-insult animal models represent another limitation. Additionally, although successful engraftment has been demonstrated previously (7–9), in-depth mechanistic validation would benefit from advanced 3D organoid platforms capable of mimicking complex epithelial–mesenchymal interactions.

## Conclusion

This study demonstrates that the hybrid combination of 2D and 3D-Festigel–cultured buccal mucosal cells provides complementary contributions to urethral epithelial repair in the BEES-HAUS technique. The 3D-Festigel cultures support epithelial regeneration, as evidenced by higher AE1/AE3 expression, whereas the 2D-cultured fibroblast-like cells secrete IGF-1, which complements the healing process thus reducing the likelihood of stricture recurrence. Transplantation using Festigel scaffold enabled effective cell delivery and engraftment in BEES-HAUS. Further investigation into such hybrid culture systems and their molecular interactions may broaden the applicability of this strategy not only for urethral reconstruction but also for regeneration of other epithelial and tissue types depending on their nature and the environment where damaged or dysfunctional cells or tissues need repair, restoration, replacement, or regeneration.

## Data Availability

The original contributions presented in the study are included in the article/**Supplementary Material**. Further inquiries can be directed to the corresponding author.

## In memoriam

The authors would like to dedicate this paper to the memory of Arokiaswamy Arokya Dass (A.A.Dass) (1944~2025) for his support to our inter-disciplinary research related overseas collaboration.

## Supplementary material

Supplementary Table 1 Comprehensive quantitative data from all 22 buccal tissue samples. The table includes (i) final cell counts following 2D monolayer and 3D Festigel culture, (ii) IGF-1 concentrations in conditioned media quantified by ELISA, and (iii) AE1/AE3 epithelial marker expression percentages determined by flow cytometry.

## References

[1] Mangir N Chapple C . Recent Advances in treatment of urethral stricture disease in men. F1000Res. (2020) 9. doi: 10.12688/f1000research.21957.1, PMID: 32419925 PMC7202089

[2] Kaluzny A Gibas A Matuszewski M . Ejaculatory disorders in men with urethral stricture and impact of urethroplasty on the ejaculatory function: A systematic review. J Sex Med. (2018) 15:974–81. doi: 10.1016/j.jsxm.2018.05.005, PMID: 29960631

[3] Rezaei M Badiei R Badiei R . The effect of platelet-rich plasma injection on post-internal urethrotomy stricture recurrence. World J Urol. (2019) 37:1959–64. doi: 10.1007/s00345-018-2597-8, PMID: 30535714

[4] Bhargava S Patterson JM Inman RD MacNeil S Chapple CR . Tissue-engineered buccal mucosa urethroplasty-clinical outcomes. Eur Urol. (2008) 53:1263–9. doi: 10.1016/j.eururo.2008.01.061, PMID: 18262717

[5] Ram-Liebig G Barbagli G Heidenreich A Fahlenkamp D Romano G Rebmann U . Results of use of tissue-engineered autologous oral mucosa graft for urethral reconstruction: A multicenter, prospective, observational trial. EBioMedicine. (2017) :23:185–192. doi: 10.1016/j.ebiom.2017.08.014, PMID: 28827035 PMC5605371

[6] Scott KA Li G Manwaring J Nikolavsky DA Fudym Y Caza T . Liquid buccal mucosa graft endoscopic urethroplasty: a validation animal study. World J Urol. (2020) 38:2139–45. doi: 10.1007/s00345-019-02840-5, PMID: 31175459

[7] Vaddi SP Reddy VB Abraham S . Buccal epithelium Expanded and Encapsulated in Scaffold-Hybrid Approach to Urethral Stricture (BEES-HAUS) procedure: A novel cell therapy-based pilot study. Int J Urol. (2018). 26(2):253–7. doi: 10.1111/iju.13852, PMID: 30468021 PMC7379713

[8] Horiguchi A Shinchi M Ojima K Hirano Y Kushibiki T Mayumi Y . Engraftment of transplanted buccal epithelial cells onto the urethrotomy site, proven immunohistochemically in rabbit model; a feat to prevent urethral stricture recurrence. Stem Cell Rev Rep. (2022). 19(1):275–8. doi: 10.1007/s12015-022-10466-1, PMID: 36306011 PMC9823073

[9] Horiguchi A Ojima K Shinchi M Kushibiki T Mayumi Y Miyai K . Successful engraftment of epithelial cells derived from autologous rabbit buccal mucosal tissue, encapsulated in a polymer scaffold in a rabbit model of a urethral stricture, transplanted using the transurethral approach. Regen. Ther. (2021) 18:127–32. doi: 10.1016/j.reth.2021.05.004, PMID: 34189194 PMC8203727

[10] Katoh S Rao SK Suryaprakash V Horiguchi A Kushibiki T Ojima K . A 3D polymer scaffold platform for enhanced *in vitro* culture of human & Rabbit buccal epithelial cells for cell therapies. Tokai J Exp Clin Med. (2021) 46:1–6. 33835468

[11] Horiguchi A Vaddi S Rajappa S Preethy S Abraham SJK . BEES-HAUS preventing urethral stricture recurrence by restoring the integrity of urothelium and its further simplified version, the BHES-HAUS. Front Bioeng Biotechnol. (2025). 13:1687741. doi: 10.3389/fbioe.2025.1687741/abstract 41394973 PMC12695836

[12] Anthony DF Shiels PG . Exploiting paracrine mechanisms of tissue regeneration to repair damaged organs. Transplant Res. (2013) 2:10. doi: 10.1186/2047-1440-2-10, PMID: 23786652 PMC3718694

[13] Foster C Jensen T Finck C Rowe CK . Development of a wound-healing protocol for *in vitro* evaluation of urothelial cell growth. Methods Protoc. (2023) 6:64. doi: 10.3390/mps6040064, PMID: 37489431 PMC10366823

[14] Shinchi M Kushibiki T Mayumi Y Ito K Asano T Ishihara M . Insulin-like growth factor 1 sustained-release collagen on urethral catheter prevents stricture after urethral injury in a rabbit model. Int J Urol. (2019) 26:572–7. doi: 10.1111/iju.13931, PMID: 30806004

[15] Terrovitis JV Smith RR Marbán E . Assessment and optimization of cell engraftment after transplantation into the heart. Circ Res. (2010) 106:479–94. doi: 10.1161/CIRCRESAHA.109.208991, PMID: 20167944 PMC2826722

[16] Horiguchi A Ojima K Shinchi M Mayumi Y Kushibiki T Katoh S . *In vitro* culture expansion and characterization of buccal mucosal epithelial cells for tissue engineering applications in urethral stricture after transportation using a thermoreversible gelation polymer. Biopreserv Biobank. (2022) 20:97–103. doi: 10.1089/bio.2021.0079, PMID: 34962137

[17] Costea DE Loro LL Dimba EA Vintermyr OK Johannessen AC . Crucial effects of fibroblasts and keratinocyte growth factor on morphogenesis of reconstituted human oral epithelium. J Invest Dermatol. (2003) 121:1479–86. doi: 10.1111/j.1523-1747.2003.12616.x, PMID: 14675199

[18] Raya-Rivera A Esquiliano DR Yoo JJ Lopez-Bayghen E Soker S Atala A . Tissue-engineered autologous urethras for patients who need reconstruction: an observational study. Lancet. (2011) 377:1175–82. doi: 10.1016/S0140-6736(10)62354-9, PMID: 21388673 PMC4005887

[19] Periasamy R Elshaer SL Gangaraju R . CD140b (PDGFRβ) signaling in adipose-derived stem cells mediates angiogenic behavior of retinal endothelial cells. Regener Eng Transl Med. (2019) 5:1–9. doi: 10.1007/s40883-018-0068-9, PMID: 30976657 PMC6453132

[20] Sudha B Madhavan HN Sitalakshmi G Malathi J Krishnakumar S Mori Y . Cultivation of human corneal limbal stem cells in Mebiol gel - A thermo-reversible gelation polymer. Indian J Med Res. (2006) 124:655–64. 17287553

[21] Parikumar P Haraguchi K Senthilkumar R Abraham SJ . Human corneal endothelial cell transplantation using nanocomposite gel sheet in bullous keratopathy. Am J Stem Cells. (2018) 7:18–24. 29531856 PMC5840311

[22] William JB Prabakaran R Ayyappan S Puskhinraj H Rao D Manjunath S Thamaraikannan P . Functional recovery of spinal cord injury following application of intralesional bone marrow mononuclear cells embedded in polymer scaffold - two year follow-up in a canine. J Stem Cell Res Ther. (2011) 1:110. doi: 10.4172/2157-7633.1000110, PMID: 39887974

[23] Parveen N Khan AA Baskar S Habeeb MA Babu R Abraham S . Intraperitoneal transplantation of hepatocytes embedded in thermoreversible gelation polymer (Mebiol gel) in acute liver failure rat model. Hepatitis Monthly. (2008) 8:275–80.

[24] Senthilkumar R Manjunath S Baskar S Dedeepiya DV Natarajan S John S . Successful Transportation and *in vitro* Expansion of Human Retinal Pigment Epithelium and its Characterization; a step towards Cell-based Therapy for Age related Macular Degeneration. Curr Trends Biotechnol Pharm. (2012) 6:44–54.

[25] Katoh S Yoshioka H Suzuki S Nakajima H Iwasaki M Senthilkumar R . An efficient polymer cocktail-based transportation method for cartilage tissue, yielding chondrocytes with enhanced hyaline cartilage expression during *in vitro* culturing. J Orthopaedics. (2022) 29:60–4. doi: 10.1016/j.jor.2022.01.007, PMID: 35145328 PMC8814592

[26] Kataoka K Huh N . Application of a thermo-reversible gelation polymer, mebiol gel, for stem cell culture and regenerative medicine. J Stem Cells Regener Med. (2010) 6:10–4. doi: 10.46582/jsrm.0601003, PMID: 24693055 PMC3908250

[27] Namitha B Chitra MR Bhavya M Parikumar P Katoh S Yoshioka H . A novel human donor cornea preservation cocktail incorporating a thermo-reversible gelation polymer (TGP), enhancing the corneal endothelial cell density maintenance and explant culture of corneal limbal cells. Biotechnol Lett. (2021) 43:1241–51. doi: 10.1007/s10529-021-03116-y, PMID: 33768381 PMC8113287

[28] Rao S Sudhakar J Parikumar P Natarajan S Insaan A Yoshioka H . Successful Transportation of Human Corneal Endothelial Tissues without Cool preservation in varying Indian Tropical climatic Conditions and *in vitro* Cell Expansion using a novel Polymer. Indian J Ophthalmol. (2014) 62:130–5. doi: 10.4103/0301-4738.116457, PMID: 24008800 PMC4005225

[29] Sitalakshmi G Sudha B Madhavan HN Vinay S Krishnakumar S Mori Y . Ex vivo cultivation of corneal limbal epithelial cells in a thermoreversible polymer (Mebiol Gel) and their transplantation in rabbits: an animal model. Tissue Eng Part A. (2009) 15:407–15. doi: 10.1089/ten.tea.2008.0041, PMID: 18724830

[30] Sankaranarayanan S Jetty N Gadagi JS Preethy S Abraham SJ . Periodontal regeneration by autologous bone marrow mononuclear cells embedded in A novel thermo reversible gelation polymer – report with 36 months follow-up. J Stem Cells. (2014) 8:99–103. 24698986

[31] Senthilkumar R Yoshioka H Katoh S Iwasaki M Surya Prakash V Balamurugan M . Engraftment and proliferation of thermoreversible-gelation-polymer-encapsulated human corneal limbal-stem-cells on ocular surface of a cadaver cornea. Curr Eye Res. (2023) 48:564–72. doi: 10.1080/02713683.2023.2180039, PMID: 36852699

[32] Ortac M Ekerhult TO Zhao W Atala A . Tissue engineering graft for urethral reconstruction: is it ready for clinical application? Urol Res Pract. (2023) 49:11–8. doi: 10.5152/tud.2023.22226, PMID: 37877833 PMC10081087

[33] Noritaka M Rosuke I Yohei M Natsuki A Masahiro M Akira M . Ureter reconstruction using a biotube in a canine model: A pilot study. J Stem Cells Regener Med. (2025). 10.46582/jsrm.2102009 PMC12766063 41497244

