# Correction: A hybrid combination of *in vitro* cultured buccal mucosal cells using two different methodologies, complementing each other in successfully repairing a stricture-inflicted human male urethral epithelium

**DOI:** 10.3389/fruro.2026.1813098

**Published:** 2026-03-26

**Authors:** Akio Horiguchi, Toshihiro Kushibiki, Yoshine Mayumi, Masayuki Shinchi, Kenichiro Ojima, Yusuke Hirano, Shojiro Katoh, Masaru Iwasaki, Surya Prakash Vaddi, Koji Ichiyama, Rajappa Senthilkumar, Senthilkumar Preethy, Samuel J. K. Abraham

**Affiliations:** 1 Division of Reconstruction, Center for Trauma, Burn and Tactical Medicine, National Defence Medical College, Tokorozawa, Saitama, Japan; 2 Department of Medical Engineering, National Defence Medical College, Tokorozawa, Saitama, Japan; 3 Department of Orthopedics, Edogawa Hospital, Edogawa, Japan; 4 Center for Advancing Clinical Research (CACR), Faculty of Medicine, University of Yamanashi, Chuo, Japan; 5 Department of Urology, Surya Kidney Centre, Hyderabad, India; 6 Department of Urology, Kamineni Academy of Medical Sciences and Research Centre, Hyderabad, India; 7 Antony- Xavier Interdisciplinary Scholastics (AXIS), GN Corporation Co. Ltd., Kofu, Japan; 8 R&D Division, JBM Inc., Edogawa, Japan; 9 The Fujio-Eiji Academic Terrain (FEAT), Nichi-In Centre for Regenerative Medicine (NCRM), Chennai, India; 10 The Mary-Yoshio Translational Hexagon (MYTH)-NCRM, MediNippon Healthcare Pvt. Ltd., Chennai, India; 11 II Department of Surgery, University of Yamanashi, Chuo, Japan; 12 Levy-Jurgen Transdisciplinary Exploratory (LJTE), Global Niche Corp., Wilmington, DE, United States; 13 Surya Akio Horiguchi Lab for Tissue Engineering (SALT), SoulSynergy Ltd., Phoenix, Mauritius

**Keywords:** BEES-HAUS, buccal mucosa, epithelium, hybrid culture, IGF-1, regenerative medicine, cell therapy, urethral stricture

## Abstract

**Figure:**
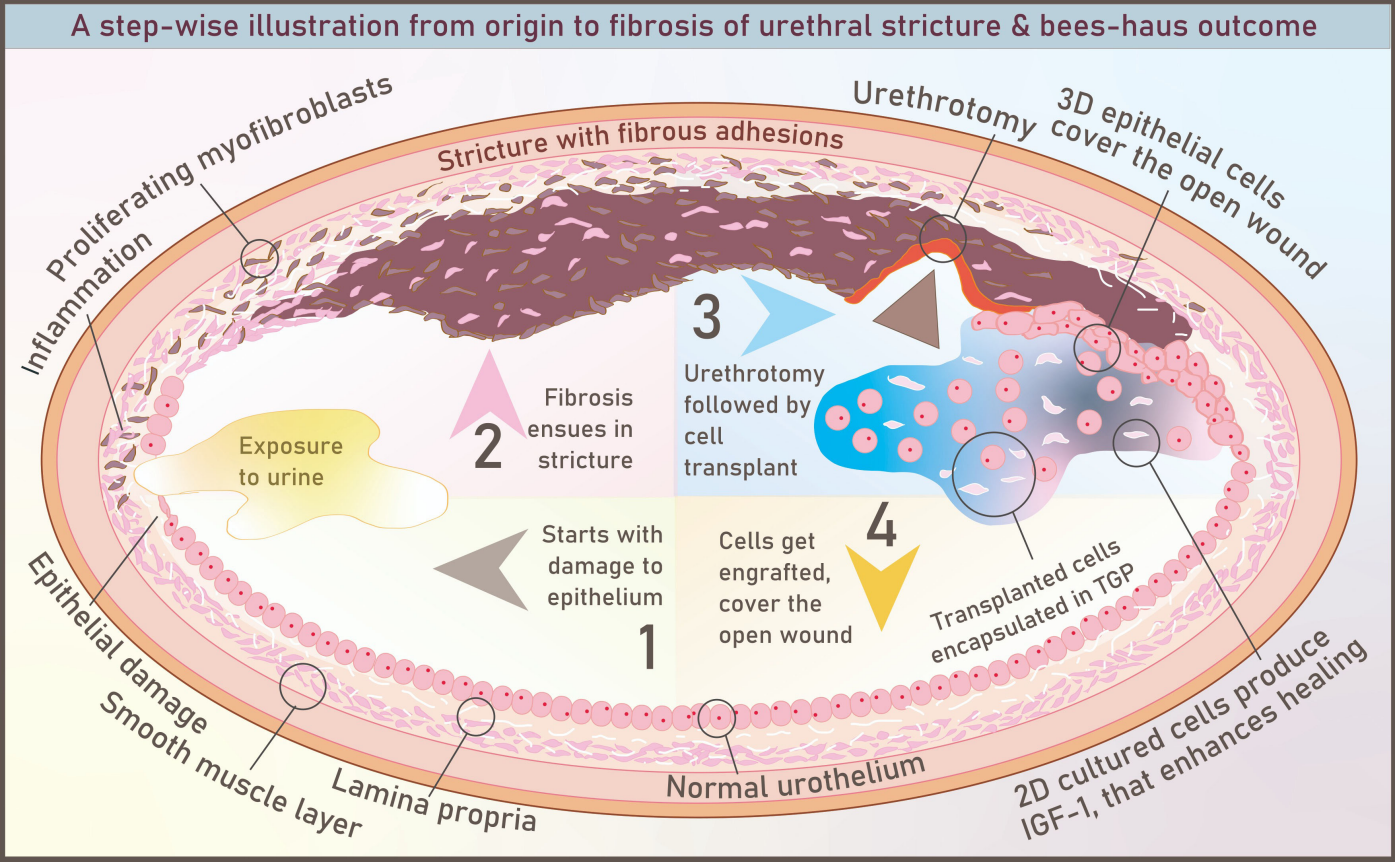

There was a mistake in **Graphical abstract** as published. The error was “Urethrotomy follows cell transplant”. The corrected form is “Urethrotomy followed by cell transplant”. The corrected figure **Graphical abstract** appears below.

The original version of this article has been updated.

