# Supplementary material for: A hybrid combination of in vitro cultured buccal mucosal cells using two different methodologies, complementing each other in successfully repairing a stricture-inflicted human male urethral epithelium
Source: Front Urol. 2026 Jan 14;5:1720445. doi: 10.3389/fruro.2025.1720445 (PMC12846977; doi:10.3389/fruro.2025.1720445)
Supplement: Supplementary Table 1 — Comprehensive quantitative data from all 22 buccal tissue samples. The table includes (i) final cell counts following 2D monolayer and 3D Festigel culture, (ii) IGF-1 concentrations in conditioned media quantified by ELISA, and (iii) AE1/AE3 epithelial marker expression percentages determined by flow cytometry. [file Table1.docx]

**Supplementary Table S1:** Comprehensive quantitative data from all 22 buccal tissue samples. The table includes (i) final cell counts following 2D monolayer and 3D Festigel culture, (ii) IGF-1 concentrations in conditioned media quantified by ELISA, and (iii) AE1/AE3 epithelial marker expression percentages determined by flow cytometry

| **Sample** | **Cell count (x 10^6^)** | | **AE1/AE3 positivity (%)** | | **IGF-1 (ng/mL)** | |
| --- | --- | --- | --- | --- | --- | --- |
|  | **2D** | **3D** | **2D** | **3D** | **2D** | **3D** |
| 1 | 0 | 0.125 | 0.35 | 4.24 | 3.18 | 2.45 |
| 2 | 0.015 | 0.33 | 0.21 | 11.8 | 4.89 | 3.08 |
| 3 | 0.009 | 0.728 | 1.1 | 15.7 | 4.73 | 3.65 |
| 4 | 1.16 | 1.2 | 0.09 | 10 | 6.06 | 2.96 |
| 5 | 0.02 | 2.67 | 1.56 | 10.3 | 5.85 | 4.13 |
| 6 | 1.14 | 1.012 | 0.02 | 16.4 | 7.82 | 6.04 |
| 7 | 0.017 | 2.14 | 8 | 5.45 | 3.68 | 2.29 |
| 8 | 0.99 | 1.67 | 6.01 | 18.6 | 4.8 | 2.27 |
| 9 | 0.81 | 1.58 | 0.24 | 12.3 | 3.85 | 2.76 |
| 10 | 3.63 | 3.62 | 0.7 | 12.1 | 7.87 | 4.35 |
| 11 | 0.63 | 1.08 | 0.23 | 7.49 | 4.12 | 3.48 |
| 12 | 1.69 | 2.36 | 0.37 | 16.9 | 2.19 | 2.16 |
| 13 | 1.2 | 2.04 | 0.1 | 24.6 | 5.65 | 2.27 |
| 14 | 2.027 | 5.34 | 0.72 | 6.67 | 3.59 | 1.89 |
| 15 | 0.607 | 1.16 | 1.9 | 19.7 | 3.39 | 1.97 |
| 16 | 0.08 | 0.23 | 0.3 | 5.19 | 3.11 | 2.39 |
| 17 | 0.26 | 3.64 | 0.26 | 4.59 | 6.64 | 5.05 |
| 18 | 0.38 | 0.86 | 0.34 | 8.49 | 3.9 | 2.63 |
| 19 | 0.36 | 5.46 | 0.12 | 15.2 | 4.32 | 2.37 |
| 20 | 0.62 | 0.84 | 0.11 | 13.4 | 3.02 | 2.62 |
| 21 | 0.16 | 0.64 | 0.05 | 13.7 | 6.01 | 4.96 |
| 22 | 0.16 | 1.94 | 0.21 | 28.9 | 3.93 | 2.94 |
